# Resource Allocation for Secure MIMO-SWIPT Systems in the Presence of Multi-Antenna Eavesdropper in Vehicular Networks

**DOI:** 10.3390/s23198069

**Published:** 2023-09-25

**Authors:** Vieeralingaam Ganapathy, Ramanathan Ramachandran, Tomoaki Ohtsuki

**Affiliations:** 1 Department of Electronics and Communication Engineering, Amrita School of Engineering, Amrita Vishwa Vidyapeetham, Coimbatore 641112, India; g_vieeralingaam@cb.students.amrita.edu (V.G.); r_ramanathan@cb.amrita.edu (R.R.); 2 Department of Information and Computer Science, Keio University, Tokyo 108-8345, Japan

**Keywords:** vehicular networks, SWIPT, MIMO, secrecy capacity, convex optimization

## Abstract

In this paper, we optimize the secrecy capacity of the legitimate user under resource allocation and security constraints for a multi-antenna environment for the simultaneous transmission of wireless information and power in a dynamic downlink scenario. We study the relationship between secrecy capacity and harvested energy in a power-splitting configuration for a nonlinear energy-harvesting model under co-located conditions. The capacity maximization problem is formulated for the vehicle-to-vehicle communication scenario. The formulated problem is non-convex NP-hard, so we reformulate it into a convex form using a divide-and-conquer approach. We obtain the optimal transmit power matrix and power-splitting ratio values that guarantee positive values of the secrecy capacity. We analyze different vehicle-to-vehicle communication settings to validate the differentiation of the proposed algorithm in maintaining both reliability and security. We also substantiate the effectiveness of the proposed approach by analyzing the trade-offs between secrecy capacity and harvested energy.

## 1. Introduction

Simultaneous wireless information and power transfer (SWIPT) is an optimistic and robust method for sustainable power supply to wireless networks [1]. The lifetime of a typical node in wireless networks can be extended by energy harvesting. Solar and RF energy are popular sources of energy in the environment. RF energy sources are used for energy harvesting to make a system independent of weather conditions. Also, the RF signals can carry information and energy simultaneously [1], hence the name SWIPT.

The challenges of controlling the data rate and desired quality of service necessitate a cooperative multiple-input multiple-output (CMIMO) system [2]. CMIMO systems can improve the performance of wireless networks and wireless sensor networks. A comprehensive overview of various supporting technologies, such as compressive sensing and SWIPT, can be augmented to CMIMO to improve the throughput and energy efficiency performance. The challenges involved in the augmented CMIMO systems, energy-saving techniques, and overview of protocol layers are analyzed in [3]. To increase the capacity and coverage of wireless networks, heterogeneous networks with macrocells and femtocells have become a popular architecture. However, the regulation of interference between these layers still poses a serious difficulty and requires efficient mitigation techniques. In [4], a joint optimization problem of maximizing the sum rates to inter-tier interference under resource allocation constraints is proposed to reduce the interference to macrocell users.

The use of different SWIPT system settings in a variety of applications, including Internet of Things (IoT) and biosensors, has prompted researchers to investigate the reliability and sustainability of the SWIPT system by analyzing the rate–energy trade-off. In the MIMO environment, the transmit power affects the performance of the SWIPT system. The performance analysis can be conducted by calculating the mean square error (MSE) of the detected/received symbol [5] and also by analyzing the relationship between the transmit power and the interference regimes present at the receiver end [6]. Since the transmit power is directly related to the information rate of a legitimate receiver, the security aspect of wireless communication systems must be analyzed. For example, the usability of MIMO systems without secure protocols will compromise the system; therefore, secure transmission algorithms in the presence of multiple eavesdroppers are studied in [7]. The keyless physical layer security (PLS) represents a subset of theoretical paradigms in wireless communications that optimize the inherent properties of the physical layer to ensure secrecy/reliability (without the need for traditional cryptographic keys). A comprehensive survey based on keyless PLS for ensuring secrecy in the wireless network environment is studied in [8], where the security challenges in IoT and V2X networks are discussed. Also in [9], a survey is conducted of the impact of security on the sixth-generation (6G) wireless technologies, network architecture and potential applications. A detailed analysis related to the security requirements, distributed machine learning (DML) is also studied.

A multi-agent deep reinforcement learning (DRL)-based approach is studied in [10] for the vehicular edge-computing network, where a secure resource allocation strategy is proposed by optimizing the transmission power, spectrum and computation resource allocation components. The DRL approach proposed in [10] significantly reduces the delay while maintaining the confidentiality probability. In the context of IoT networks, work in [11] explores the cooperative potential of non-orthogonal multiple access (NOMA) for SWIPT, addressing the challenges of imperfect successive interference cancellation (SIC) and using deep learning to optimize the throughput performance. In [12], a novel semi-supervised intrusion detection method based on federated learning is proposed to improve the quality of the predicted outputs (thereby avoiding incorrect predictions and achieving lower communication overhead). The recent work proposed in [13] explores the potential of deep learning applications in device-to-device (D2D) unmanned aerial vehicle (UAV) communication, leveraging the use of both SWIPT and multi-agent deep Q-networks (MADQN) to improve energy efficiency based on the design of reward functions. In the context of the rapidly evolving and highly dynamic vehicular ad hoc networks (VANETs) landscape, as studied in [14], the deep neural network framework for anomaly detection contributes to jointly enhance the security and reliability of these networked systems.

Dynamic settings and the mobility of devices in wireless network environments (such as unmanned aerial vehicle (UAV) and ad hoc networks) present additional difficulties and opportunities for ensuring effective and reliable data transmission and resource management. Vehicle clustering provides a solution for dynamic wireless networks, enabling effective resource management and solving mobility issues. For ad hoc networks, the vehicle-clustering algorithm plays a key role in establishing effective communication and resource sharing among vehicles. In [15], a clustering algorithm is proposed to minimize the total energy consumption of vehicles based on their direction and entropy using a fuzzy C-means algorithm. As discussed in [16], three-dimensional trajectory planning in UAV networks is crucial to enable seamless cooperation and data exchange/collection among UAVs, optimize their flight paths, and efficiently utilize scarce resources.

The transmit power, along with the number of antennas, plays a significant role in determining the secrecy rate of the system. Due to the characteristics of RF signals in SWIPT systems, separate and co-located energy-harvesting/information-decoding (EH/ID) receivers with corresponding precoder designs are being developed [17]. The characterization of different capacity scaling techniques (in general, the capacity values vary depending on the base station (BS) antennas, signal-to-noise ratio (SNR), beamforming architecture, and coherence block size) are investigated for the massive MIMO systems under strong spatial correlation regimes as shown in [18]. Based on the optimization of transmit beamforming vectors and power-splitting ratios, the battery depletion phenomenon is mitigated by formulating an optimal resource allocation strategy based on the transmit power optimization algorithm [19]. The channel characteristics affect the received energy in a wireless network, thus affecting the energy-harvesting capability of the intended receiver. The amount of energy harvested for passively powered sensor networks is investigated in [20], where the circuit is designed and relationships between the DC power and received power are analyzed.

Vehicular networks are becoming increasingly interconnected and autonomous, leading to the need for reliable, secure, and sustainable communication mechanisms. Recent research has shown that integrating SWIPT into vehicular networks offers promising solutions to energy sustainability concerns [21,22]. As relays increasingly become “hubs”—collecting, sending, and receiving large amounts of information—the energy and security requirements of in-vehicle communication systems are increased dramatically [23]. Integrating vehicle networks with SWIPT promises a dual benefit: reliable communications, and reduced dependence on external power sources. This synergy of security and energy harvesting can significantly increase the potential autonomy of vehicular communication systems. The impact of distance and control over the power-splitting factor on physical layer security is studied in [24]. The authors also investigated jamming techniques to improve security (as the distance between jammer and eavesdropper changes).

### 1.1. Motivation

The presence of a sophisticated eavesdropper equipped with multiple antennas adds another layer of complexity. Multi-antenna eavesdroppers can intercept signals from different spatial paths, posing a significant threat to MIMO communications and SWIPT systems. A nuanced approach to resource allocation is essential to ensure both efficient energy harvesting and secure data transmission in such scenarios. Our work aims to address the overlapping challenges of energy and PLS management in vehicular networks, laying the foundation for a more resilient and sustainable vehicular communication system. With the significant modernization and increased applicability of MIMO-SWIPT vehicular networks in industrial applications, ensuring secure communication in the presence of potential eavesdroppers becomes a paramount concern. The potential vulnerabilities posed by multi-antenna eavesdroppers in such vehicular networks necessitate the development of robust resource allocation strategies to secure wireless power and information transmission. By optimizing the transmit power allocation, our work aims to enhance the secrecy capacity and harvested energy in MIMO-SWIPT systems, thus ensuring reliable and secure communication for vehicular applications. Our research aims to contribute to the advancement of secure resource allocation techniques, enabling sustainable and secure wireless communication integration in vehicular networks while addressing security challenges in dynamic communication environments.

### 1.2. Contributions

In this paper, we present a resource allocation strategy for a MIMO SWIPT system in vehicular networks to optimize the information capacity of multi-antenna Bob under resource allocation constraints in the presence of a multi-antenna passive eavesdropper. The information capacity maximization problem with secrecy rate and quality-of-service constraints is formulated for different positions of Bob, and transmit power/power-splitting (PS) ratio values are achieved using the divide-and-conquer strategy. The following dynamic scenario is considered: Bob is moving from position-A (Pos-A) to position-B (Pos-B). Also, Pos-B is further away from Alice (compared to Pos-A) because Bob traverses from Pos-A to Pos-B away from Alice in the presence of a multi-antenna eavesdropper (which is positioned relatively close to Alice). The contributions of this paper are as follows:

- We present two transmission scenarios in SWIPT-enabled vehicular networks in the presence of a multi-antenna eavesdropper: Bob is at Pos-A, and Bob is at Pos-B.
- We formulate corresponding optimization problems for two scenarios: when Bob is at Pos-A and when Bob is at Pos-B under resource allocation constraints.
- We maximize the information capacity under the secrecy capacity, harvested energy requirements, and other resource allocation constraints.
- We propose a divide-and-conquer strategy by splitting the optimization problem into two sub-problems (alternating the choice of optimization variables). By optimizing the power-splitting ratio, our proposed algorithm can allocate the harvested energy to the EH/ID receiver and maintain sufficient secrecy capacity.
- We provide simulation results, highlighting the differences between the transmission scenarios considered. We also validate the need for the proposed scheme by analyzing the performance under the two considered scenarios.

### 1.3. Related Work

The transmit power parameter is one of the key variables in determining the information rate for wireless communication systems. The transmit power can be optimized by using beamforming resource allocation strategies. Since we incorporated SWIPT in the MIMO system, there is also a need to optimize the value of the power-splitting ratio. The system is modeled without the inclusion of SWIPT, and is studied in the works [6,18], where the beamforming problem for cognitive radio and MIMO environments is developed. In [6], the transmit power for secondary users is minimized, while the power for the primary user is increased. The problem is reformulated as a relaxed semidefinite problem, and transmit power/SNR analysis is performed for primary and secondary users.

In [18], the achievable rate in a MIMO environment is analyzed. The rate-scaling characteristics are studied for different values of antennas. The analytical structure of the information rate depends on the selected channel characteristics. An investigation of the use of multiple transmit/receive antennas for a single-user communication model is studied for fading and non-fading channels [25]. The capacities and error exponents of both fading and non-fading channels are formulated. The result shows that the use of multiple antennas significantly increases the information capacity of a single user.

By incorporating a SWIPT perspective into the wireless communication model, the rate–energy (R-E) characteristics are analyzed as in [1,3], and the corresponding beamforming vector optimization strategy is used to improve the efficiency (by preventing depletion and ensuring the latency/harvesting required amount of energy) as in [19]. The characteristics of EH/ID receivers are studied for various practical designs. The co-located and separated EH/ID receivers and their relationship with information rates are analyzed. The reliability of the practical parameters present in the receiver circuit must be introspected and incorporated in the study of the rate–energy trade-off. The effect of the circuit specification on the R-E region is analyzed in [3]. A multi-antenna SWIPT framework is studied in [19], where both (i) the semi-definite relaxation (SDR) approach combined with fractional programming (FP), and (ii) successive convex approximation (SCA) are proposed to achieve a robust and energy-efficient resource allocation strategy to mitigate battery depletion.

The hardware imperfections and distortions occur due to imbalance and nonlinearity in the high-power amplifier (HPA) and phase noise, which can degrade the quality of the EH/ID receivers. In [26], the estimation of distortions present in a SWIPT power-splitting system is investigated. The nonlinearity and total harmonic distortion present in the output of the HPA are analyzed using the recursive least squares technique. An analysis of the received SNR and harvested energy is performed for different harmonic distortion characteristics. In [27], a PS-based SWIPT system is studied under hardware impairments and in-phase and quadrature imbalances. An optimization problem is proposed to maximize the harvested energy under a SNR constraint in the presence of hardware impairments. An optimal value of harvested energy is achieved using bio-inspired algorithms in a Rayleigh fading environment.

The energy-harvesting capability can also cause interference in the communication environment, so the selection of optimal users based on a resource allocation strategy is developed in [28]. The use and design of appropriate variables in the resource allocation algorithms guarantee the selection of sensors and users depending on the defined optimization problem under the energy-harvesting wireless network environment [28].

The number of deployable nodes and transmission strategies is increasing in a wireless network environment, so several relay selection strategies need to be explored. In [29], a novel and efficient relay node selection scheme based on a power-splitting ratio for decoding and forward cooperative relay networks under the SWIPT framework is proposed. A power-splitting scheme is formulated to express the rate–energy exchange for the energy used for information decoding. The expression for the probability of failure is also derived, and a performance metric is analyzed in terms of energy conversion efficiency and SNR at different thresholds of the data rates.

The secrecy rate of the system is analyzed as a min-max problem for different ranges of SNR [30] in the presence of an active eavesdropper attack. The effect of multiple antennas between the sender, receiver, and eavesdropper on the secrecy rate is also analyzed. A trade-off between the average harvested energy and secrecy rate is analyzed for secure SWIPT in cell-free MIMO systems with multiple access points (APs) transmitting information to users. In [31], the authors extensively analyze the phenomenon of active pilot attack (by eavesdroppers) to compromise the base station channel and propose a methodology to achieve a secure link in intelligent reflecting surface (IRS)-assisted MIMO systems. The authors also successfully suppress the active eavesdropper using beamforming optimization (which is also validated in the trade-off curve between the secrecy capacity and eavesdropper position).

To achieve a secure wireless communication environment, a cognitive radio transmission system for decode and forward UAV with energy harvesting at the source and relay nodes is proposed [32]. The distribution of the probability of the non-zero secrecy rate of the system under the time-shared protocol is studied. A performance analysis based on the optimal secrecy rate selection strategy and the optimal antenna selection strategy at the destination node of the secondary network is investigated. The resource allocation algorithm of the SWIPT system can be extended to the security aspect of the algorithm by incorporating an analysis involving two significant characteristics: secrecy throughput, and transmission schemes, such as delay-constrained transmission and delay-tolerant transmission [33].

A resource allocation strategy is proposed to achieve a secure information rate and green power transmission for mobile receivers with distributed antennas connected to a central processor [34]. The total network transmission power is minimized under quality-of-service constraints. An optimal iterative algorithm based on generalized Bender’s decomposition is proposed.

Most practical applications require the optimization of rate and energy variables, so such variants of optimization problems are biconvex. An extensive survey of the theory of biconvex sets is given in [35], and biconvex optimization problems are solved by exploiting the properties of biconvex sets. The mathematical approach of convex optimization always aims to find optimal or suboptimal values of the variables under consideration. The penalty convex–concave approach is used to perform first-order convex approximation to solve the bilinear difference of convex problem [5]. The SDR approach for the co-located receiver is developed to achieve optimal parameter values [17].

As the technology evolves, the simulation of MIMO wireless environments requires an accurate, practical model for arriving at an optimal solution, so the nonlinear EH models are studied in [3]. The various aspects of resource allocation algorithms are developed to analyze the characteristics of MIMO system models. Resource allocation problems based on the system models provide an optimal solution to several key variables. This also helps to understand the relationship between these variables. As the deployability of SWIPT systems increases, there is a need to characterize the trade-off between the information and energy-harvesting parameters, which provides a detailed analysis on the SWIPT, WPT-enabled systems [1,3].

In summary, vehicular networks require a high rate of information exchange for synchronous and concurrent operations. Wireless networks are also growing in almost all practical aspects, so MIMO-based capabilities are being used to enhance various physical layer aspects of these systems. This use of MIMO technologies and the increasing robust use of RF signals have led to the development of SWIPT-enabled MIMO systems. From the SWIPT perspective of the wireless system, the receiver is required to maintain an optimal amount of energy-harvesting and information-decoding capability, as this feature determines the lifetime and reliability of the entire transmission.

*To the best of the authors’ knowledge, the study of optimization problems under different transmission scenarios for the MIMO-SWIPT system in the presence of a multi-antenna eavesdropper has not been studied.* To elaborate the specific novelty of our work, we compared it with the previous works. The main differences are as follows:

- In [3], the main objective is to maximize the harvested energy for time-switching and power-splitting scenarios for the MIMO broadcasting system. The authors analyzed the trade-off between information capacity and energy for separated and co-located SWIPT receivers. In our proposed work, we included the maximization of the information capacity of Bob with the co-located SWIPT system in the presence of a multi-antenna eavesdropper. Our work focuses on the secrecy capacity–harvested energy trade-off under dynamic vehicular settings.
- In [30], the authors consider active eavesdropping scenarios and solve the maximized ergodic secrecy capacity problem using semidefinite relaxation programming. We considered dynamic MIMO-SWIPT settings in the presence of multi-antenna eavesdroppers under a worst-case scenario when Bob traverses from Pos-A to Pos-B (hence different from [30]).
- In [36], the authors propose a joint channel estimation and transmit power allocation strategy to maximize the average signal-to-error-plus-noise ratio (SENR) under resource allocation constraints. The authors use a Karush–Khun–Tucker (KKT)-based solution to arrive at the solution. Unlike [36], we proposed a “divide-and-conquer strategy” by reformulating the non-convex problem into convex sub-problems based on maximizing the information capacity in the presence of the secrecy capacity and other resource allocation constraints under dynamic vehicular environment.

The rest of the paper is organized as follows. Section 2 presents the problem formulation, where the system model for the MIMO-SWIPT system in the presence of a multi-antenna eavesdropper and the problem definition for both transmission scenarios are discussed. Section 3 presents the proposed solution for both transmission scenarios and a description of the algorithm. Section 4 describes the result obtained for both transmission scenarios. The complexity analysis is also presented in this section. Section 5 concludes the paper.

## 2. Problem Formulation

This section presents the system model and problem definition for two of the transmission modes, formulating the maximization of the information capacity under the secrecy capacity, harvested energy requirements, and other resource allocation constraints.

### 2.1. System Model

**Figure 1:**
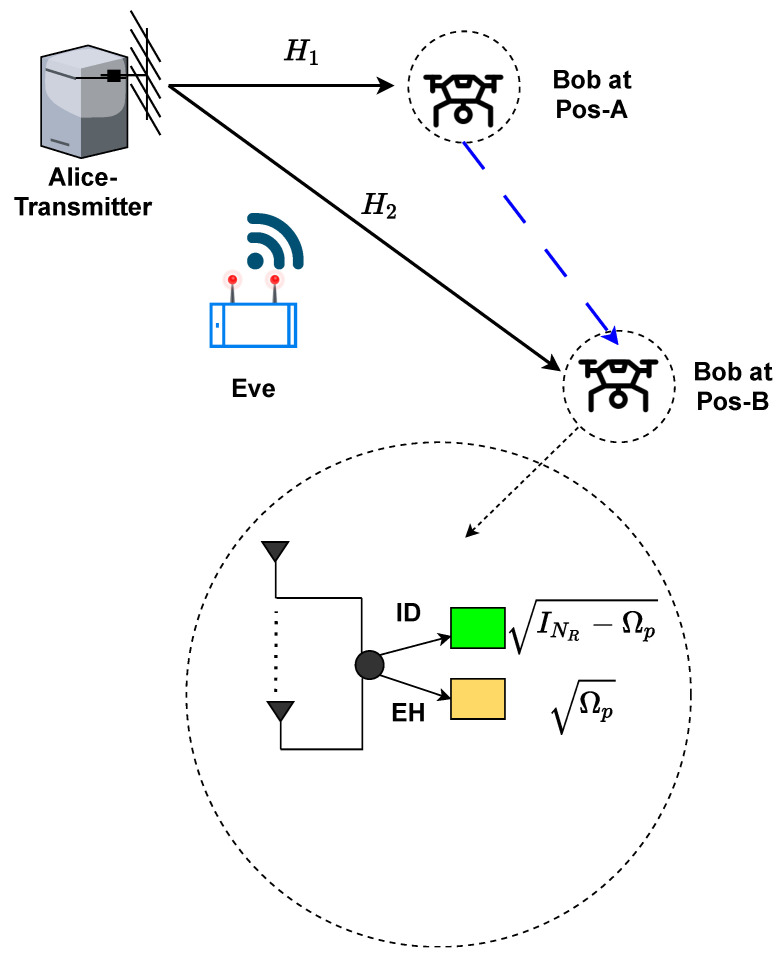
System model: In the downlink transmission mode, Alice transmits information and energy signal to multi-antenna co-located EH/ID legitimate receiver (called Bob). Multi-antenna eavesdropper attempts to tap information from the Alice as the Bob moves from position A to B.

The system model of a dynamic SWIPT-based MIMO for a co-located EH/ID receiver for a power-splitting architecture in the presence of a multi-antenna eavesdropper is considered as shown in Figure 1. We consider a multi-antenna transmitter (Alice) with multiple antennas and a co-located energy-harvesting/information-decoding (EH/ID) receiver with multiple antennas in the presence of a multi-antenna passive eavesdropper (Eve) with the same number of antennas as the receiver. The number of antennas at the transmitter is $N_{T}$, and the numbers of antennas at the receiver and eavesdropper are $N_{R}$ and $N_{E}$, respectively. Let $H_{1}$∈$C^{N_{R}\times N_{T}}$ be the channel matrix between Alice and Bob at position-A. When Bob moves from position-A (Pos-A) to position B (Pos-B), the channel fading state changes from $H_{1}$ to $H_{2}$. Let *X*$\in C^{N_{T}\times N_{R}}$ be the transmitted signal from Alice. The covariance matrix of *X* is given by $E\left[XX^{H}\right]=\mathrm{Tr}\left(Q\right)$, where $H_{E}^{1}$ and $H_{E}^{2}$ (∈$C^{N_{E}\times N_{T}}$) denote the channel matrices between Alice and the eavesdropper (Eve), while Bob is at Pos-A and Pos-B, respectively. It is assumed that Eve is close to Alice.

We consider a large-scale fading model where $D_{i}$ is given by ${\left(\frac{d_{i}}{d_{0}}\right)}^{-\alpha }$. Here, $d_{i}$ is the distance between Alice and a corresponding receiver (Bob or Eve). We consider the reference distance $d_{0}$ to be 10 m. The variable α denotes the path loss exponent with α (we take it to be three). So, we have $D_{A}^{s}={\left(\frac{d_{A}^{s}}{d_{0}}\right)}^{-\alpha }$ for Alice-[Bob at Pos-A]; $D_{B}^{s}={\left(\frac{d_{B}^{s}}{d_{0}}\right)}^{-\alpha }$ for Alice-[Bob at Pos-B]; and $D_{E}={\left(\frac{d_{E}}{d_{0}}\right)}^{-\alpha }$ for Alice-Eve. Using $D_{A}^{s}$ (for $H_{1}$), $D_{B}^{s}$ (for $H_{2}$), and $D_{E}$ (for $H_{E}^{1}\;\&\;H_{E}^{2}$), small-scale fading is incorporated using both line-of-sight and non-line-of-sight (LOS and NLOS) components. Since Bob traverses from Pos-A to Pos-B, and Pos-B is further away from Alice (than Pos-A), the value of $d_{B}^{s}$ is always greater than $d_{A}^{s}$.

### 2.2. Problem Definition

We aim to optimize the transmit power matrix contained in the information rate maximization problem for Bob at Pos-A in the presence of an eavesdropper. We derive a closed-form expression for obtaining the energy-harvesting values based on the nonlinear EH models and the channel fading state $H_{1}$. Theoretically, the information rate for a SWIPT system depends on the parameters: transmit power and power sharing ratio [3]. We formulate an information rate maximization problem under transmit power and secrecy capacity constraints. The received signal is modeled as a function of the power-splitting ratio $\left(\sqrt{\Omega_{p}}\right)$ and thus determines the value of the transmit power matrix, which is used to arrive at a harvested energy value. The term “$\left(\sqrt{I_{N_{R}}-\Omega_{p}}\right)$” in (2) indicates the received signal allocated for information decoding (since we considered the co-located SWIPT system). Theoretically, $I_{N_{R}}$ is the upper bound for the power-splitting ratio, forming an identity matrix of dimension $N_{R}\times N_{R}$. If $\sqrt{\Omega_{p}}$ is allotted for energy harvesting, then the remaining $\left(\sqrt{I_{N_{R}}-\Omega_{p}}\right)$ is naturally allotted for information decoding. The received signals at the EH $Y_{E}$ and ID $Y_{I}$ receivers can be given by

(1) $$Y_{E}^{1}=\left(\sqrt{\Omega_{p}}\right)H_{1}X,$$

(2) $$Y_{I}^{1}=\left(\sqrt{I_{N_{R}}-\Omega_{p}}\right)H_{1}X+N_{I}^{1},$$

where $N_{I}^{1}$∈$C^{N_{R}\times N_{R}}$∼$CN(0,\hat{\sigma_{i}^{2}}I_{N_{R}})$ is an additive complex Gaussian noise received at the ID portion of Bob at Pos-A. Let $\rho_{i}$ denote the power-splitting ratio for the *i*-th receiver antenna, defining $\left(\Omega_{p}\right)$ as diag$\left(\rho_{1},\rho_{2},\cdots ,\rho_{N_{R}}\right)$, 0 ≤ $\rho_{i}$ ≤ 1, where *i* varies from 1 to $N_{R}$. The received signal at the eavesdropper side $Y_{eve}$ is given by

(3) $$Y_{eve}^{1}=H_{E}^{1}X+N_{eve}^{1},$$

where $N_{eve}^{1}$∈$C^{N_{E}\times N_{E}}$∼$CN(0,\hat{\sigma^{2}}I_{N_{E}})$ is also an additive complex Gaussian noise at Eve. Similarly, the received signals at Bob (when he is at Pos-B) are given by

(4) $$Y_{E}^{2}=\left(\sqrt{\Omega_{p}}\right)H_{2}X,$$

(5) $$Y_{I}^{2}=\left(\sqrt{I_{N_{R}}-\Omega_{p}}\right)H_{2}X+N_{I}^{2}.$$

where $N_{I}^{2}$∈$C^{N_{R}\times N_{R}}$∼$CN(0,\tilde{\sigma_{i}^{2}}I_{N_{R}})$ is the additive complex Gaussian noise received at the ID portion of Bob at Pos-A. Here, the noise between Alice and the corresponding EH/ID node is varied as the position is changed. The received signal at Eve’s side $Y_{eve}^{2}$ is given by

(6) $$Y_{eve}^{2}=H_{E}^{2}X+N_{eve}^{2}.$$

where $N_{eve}^{2}$∈$C^{N_{E}\times N_{E}}$∼$CN(0,\tilde{\sigma^{2}}I_{N_{E}})$ is also an additive complex Gaussian noise at Eve. The optimization problem is formulated to maximize the information rate under the constraints of transmit power and secrecy capacity for Bob at Pos-A. The P1−A problem is given by

(7) $$\begin{array}{c} P1-A:\max_{Q,\Omega_{p}}\log_{2}∥I_{N_{R}}+\frac{{\left(I_{N_{R}}-\Omega_{p}\right)}^{\frac{1}{2}}H_{1}QH_{1}^{H}{\left(I_{N_{R}}-\Omega_{p}\right)}^{\frac{1}{2}}}{\hat{\sigma_{i}^{2}}}∥\\ C1:\mathrm{Tr}(Q)\leq P,\\ C2:0≼\Omega_{p}≼I_{N_{R}},\\ C3:Q≽0,\\ C4:\log_{2}∥I_{N_{R}}+\frac{{\left(I_{N_{R}}-\Omega_{p}\right)}^{\frac{1}{2}}H_{1}QH_{1}^{H}{\left(I_{N_{R}}-\Omega_{p}\right)}^{\frac{1}{2}}}{\hat{\sigma_{i}^{2}}}∥\\ -\log_{2}∥I_{N_{E}}+\frac{H_{E}^{1}Q{H_{E}^{1}}^{H}}{\hat{\sigma^{2}}}∥\geq R,\\ C5:\mathrm{Tr}\left(\Omega_{p}H_{1}QH_{1}^{H}\right)\geq E. \end{array}$$

Constraint *C*1 guarantees an upper bound on the value of the transmit power available in the environment. The power-splitting ratio is also bounded between zero and one to determine the distribution of the received signal for energy harvesting or information decoding. Constraint *C*3 is included to guarantee a positive semidefinite property for the transmit power matrix *Q*. Constraint *C*4 is the secrecy capacity constraint that guarantees the secure transmission of information. Constraint *C*5 is included to satisfy the harvested energy requirement *E*. When Bob is at Pos-B (which is further away from Alice compared to Pos-A), the optimization problem is modified to include the channel fading $H_{2}$, so we have a problem P2−A, where $H_{1}$ is replaced by $H_{2}$ and $H_{E}^{1}$ is replaced by $H_{E}^{2}$. The noise between Alice–Eve and Alice–Bob at Pos-B also changes. The optimization problem for Bob at Pos-B is given by

(8) $$\begin{array}{c} P1-B:\max_{Q,\Omega_{p}}\log_{2}∥I_{N_{R}}+\frac{{\left(I_{N_{R}}-\Omega_{p}\right)}^{\frac{1}{2}}H_{2}QH_{2}^{H}{\left(I_{N_{R}}-\Omega_{p}\right)}^{\frac{1}{2}}}{\tilde{\sigma_{i}^{2}}}∥\\ C1:\mathrm{Tr}(Q)\leq P,\\ C2:0≼\Omega_{p}≼I_{N_{R}},\\ C3:Q≽0,\\ C4:\log_{2}∥I_{N_{R}}+\frac{{\left(I_{N_{R}}-\Omega_{p}\right)}^{\frac{1}{2}}H_{2}QH_{2}^{H}{\left(I_{N_{R}}-\Omega_{p}\right)}^{\frac{1}{2}}}{\tilde{\sigma_{i}^{2}}}∥\\ -\log_{2}∥I_{N_{E}}+\frac{H_{E}^{2}Q{H_{E}^{2}}^{H}}{\tilde{\sigma^{2}}}∥\geq R,\\ C5:\mathrm{Tr}\left(\Omega_{p}H_{2}QH_{2}^{H}\right)\geq E. \end{array}$$

Although the denotation of the transmit power matrix *Q* remains the same, we may obtain different values of *Q* due to the change in channel fading states (and also due to the need to maintain positive secrecy).

## 3. Proposed Solution

In this section, we derive the solution to the formulated optimization problems and obtain the optimal values of transmit power matrices and power-splitting ratios for the corresponding transmission modes.

### 3.1. Transmission Mode Pos-A

The problem P1−A (7) is a non-convex NP-hard problem due to its dependence on the transmit power matrix and the power splitting ratio. Also, the problem *P*1 is in the form of a product of variables, which requires a reformulation strategy with appropriate constraints to obtain an optimal solution. The problem *P*1 has two variables $\Omega_{p}$ and *Q*, by transforming the problem into two sub-problems [35]: one sub-problem P2−A to solve *Q* for a given value of $\Omega_{p}$ and another sub-problem P3−A to solve $\Omega_{p}$ for a given value of *Q*. We guarantee optimal values of the transmit power matrix *Q* and $\Omega_{p}$.

#### 3.1.1. Sub-Problem P2

Keeping the transmit power matrix *Q* as the only variable, the subproblem $\hat{P2}-A$ is given by

(9) $$\begin{array}{c} \hat{P2}-A:\max_{Q}\log_{2}∥I_{N_{R}}+\frac{{\left(I_{N_{R}}-\Omega_{p}\right)}^{\frac{1}{2}}H_{1}QH_{1}^{H}{\left(I_{N_{R}}-\Omega_{p}\right)}^{\frac{1}{2}}}{\hat{\sigma_{i}^{2}}}∥\\ C1:\mathrm{Tr}(Q)\leq P,\\ C2:Q≽0,\\ C3:\log_{2}∥I_{N_{R}}+\frac{{\left(I_{N_{R}}-\Omega_{p}\right)}^{\frac{1}{2}}H_{1}QH_{1}^{H}{\left(I_{N_{R}}-\Omega_{p}\right)}^{\frac{1}{2}}}{\hat{\sigma_{i}^{2}}}∥\\ -\log_{2}∥I_{N_{E}}+\frac{H_{E}^{1}Q{H_{E}^{1}}^{H}}{\hat{\sigma^{2}}}∥\geq R,\\ C4:\mathrm{Tr}\left(\Omega_{p}H_{1}QH_{1}^{H}\right)\geq E. \end{array}$$

Here, the constraint C3 is in the form of a difference of concave functions and must be reformulated. Let

(10) $$I_{N_{R}}+\frac{{\left(I_{N_{R}}-\Omega_{p}\right)}^{\frac{1}{2}}H_{1}QH_{1}^{H}{\left(I_{N_{R}}-\Omega_{p}\right)}^{\frac{1}{2}}}{\hat{\sigma_{i}^{2}}}≜R_{\Omega },$$

(11) $$I_{N_{E}}+\frac{H_{E}^{1}Q{H_{E}^{1}}^{H}}{\hat{\sigma^{2}}}≜R_{E}.$$

From (10), we obtain

(12) $$H_{1}QH_{1}^{H}={\left({\left(I_{N_{R}}-\Omega_{p}\right)}^{\frac{1}{2}}\right)}^{-1}\left(\hat{\sigma_{i}^{2}}\left(R_{\Omega }-I_{N_{R}}\right)\right){\left({\left(I_{N_{R}}-\Omega_{p}\right)}^{\frac{1}{2}}\right)}^{-1},$$

Let $T_{EH}$ be the variable denoting harvested energy. After incorporating (12), we have

(13) $$T_{EH}=\mathrm{Tr}\left(\Omega_{p}{\left({\left(I_{N_{R}}-\Omega_{p}\right)}^{\frac{1}{2}}\right)}^{-1}\left(\hat{\sigma_{i}^{2}}\left(R_{\Omega }-I_{N_{R}}\right)\right){\left({\left(I_{N_{R}}-\Omega_{p}\right)}^{\frac{1}{2}}\right)}^{-1}\right).$$

Also, (11) can be rewritten as

(14) $$Q={\left(H_{E}^{1}\right)}^{†}\left(\hat{\sigma^{2}}\left(R_{E}-I_{N_{E}}\right)\right){\left({H_{E}^{1}}^{H}\right)}^{†}.$$

The inverse of a non-square matrix requires the use of a pseudoinverse or Moore–Penrose inverse. Thus, the inverse of $H_{E}^{1}$ is represented by ${\left(H_{E}^{1}\right)}^{†}$ (we can only use ${\left(H_{E}^{1}\right)}^{-1}$ if $H_{E}^{1}$ is a square matrix). The Moore–Penrose inverse of ${\left(H_{E}^{1}\right)}^{†}$ is given by ${\left(H_{E}^{1}\right)}^{H}{\left(H_{E}^{1}{\left(H_{E}^{1}\right)}^{H}\right)}^{-1}$. Using (10) and (11), the constraint C3 from the subproblem $\tilde{P2}-A$ can be rewritten as

(15) $$\log_{2}∥R_{\Omega }∥-\log_{2}∥R_{E}∥\geq R.$$

The first-order Taylor approximation is performed on the *C*3 [37], and *C*3 is modified into

(16) $$\begin{array}{c} \log_{2}∥R_{\Omega }∥-\log_{2}∥R_{E}∥\approx \log_{2}∥R_{\Omega }^{*}∥\\ +\mathrm{Tr}({\left(R_{\Omega }^{*}\right)}^{-1}\left(R_{\Omega }-R_{\Omega }^{*}\right))\\ -\log_{2}∥R_{E}^{*}∥-\mathrm{Tr}\left({\left(R_{E}^{*}\right)}^{-1}\left(R_{E}-R_{E}^{*}\right)\right). \end{array}$$

Since $\log∥R_{\Omega }∥$ is concave on $R_{\Omega }$ ≥ 0, the approximation symbol ≈ can be replaced by ≤. Using the same simplifications, the subproblem can be rewritten as $\hat{P2}-A$ as follows:

(17) $$\begin{array}{c} P2-A:\max_{R_{\Omega },R_{E}}\log_{2}∥R_{\Omega }∥\\ C1:\mathrm{Tr}({\left(H_{E}^{1}\right)}^{†}\left(\sigma^{2}\left(R_{E}-I_{N_{E}}\right)\right){\left({H_{E}^{1}}^{H}\right)}^{†})\leq P,\\ C2:R_{E}≽I_{N_{E}},\\ C3:\log_{2}∥R_{\Omega }^{*}∥+\mathrm{Tr}\left({\left(R_{\Omega }^{*}\right)}^{-1}\left(R_{\Omega }-R_{\Omega }^{*}\right)\right)\\ -\log_{2}∥R_{E}^{*}∥-\mathrm{Tr}\left({\left(R_{E}^{*}\right)}^{-1}\left(R_{E}-R_{E}^{*}\right)\right)\geq R,\\ C4:T_{EH}\geq E. \end{array}$$

The use of the solution variable $R_{E}$ from the problem P2−A (17) is substituted in (14) to obtain the intermediate transmit power matrix $Q^{\#}$. The transmit power matrix $Q^{\#}$ (computed from (14)) is used in the following subproblems to obtain the final solution.

#### 3.1.2. Sub-Problem P3

Keeping the power-splitting ratio $\Omega_{p}$ as the only variable, the subproblem $\hat{P3}-A$ is given by,

(18) $$\begin{array}{c} \hat{P3}-A:\max_{\Omega_{p}}\log_{2}∥I_{N_{R}}+\frac{{\left(I_{N_{R}}-\Omega_{p}\right)}^{\frac{1}{2}}H_{1}Q^{\#}H_{1}^{H}{\left(I_{N_{R}}-\Omega_{p}\right)}^{\frac{1}{2}}}{\hat{\sigma_{i}^{2}}}∥\\ C1:0≼\Omega_{p}≼I_{N_{R}},\\ C2:\log_{2}∥I_{N_{R}}+\frac{{\left(I_{N_{R}}-\Omega_{p}\right)}^{\frac{1}{2}}H_{1}Q^{\#}H_{1}^{H}{\left(I_{N_{R}}-\Omega_{p}\right)}^{\frac{1}{2}}}{\hat{\sigma_{i}^{2}}}∥\\ -\log_{2}∥I_{N_{E}}+\frac{H_{E}^{1}Q^{\#}{H_{E}^{1}}^{H}}{\sigma^{2}}∥\geq R,\\ C3:\mathrm{Tr}\left(\Omega_{p}H_{1}Q^{\#}H_{1}^{H}\right)\geq E. \end{array}$$

where $Q^{\#}$ is a constant, and $\Omega_{p}$ is a variable. The objective function of the subproblem $\hat{P3}-A$ can be expressed as

(19) $$W_{\Omega }≜I_{N_{R}}+\frac{{\left(I_{N_{R}}-\Omega_{p}\right)}^{\frac{1}{2}}H_{1}Q^{\#}H_{1}^{H}{\left(I_{N_{R}}-\Omega_{p}\right)}^{\frac{1}{2}}}{\hat{\sigma_{i}^{2}}}.$$

Here, $W_{\Omega }$ is a function that depends on the variable $\Omega_{p}$. Similarly, in the case of Eve, we can express the information rate between Alice and Eve as

(20) $$W_{E}≜I_{N_{E}}+\frac{H_{E}^{1}Q^{\#}{H_{E}^{1}}^{H}}{\hat{\sigma^{2}}}.$$

The variables $W_{\Omega }$ and $W_{E}$ are positive semidefinite matrices, and the subproblem $\hat{P3}-A$ (18) can be recast into

(21) $$\begin{array}{c} P3-A:\max_{W_{\Omega },W_{E},\Omega_{p}}\log_{2}∥W_{\Omega }∥\\ C1:W_{\Omega }≽0,\\ C2:W_{E}≽0,\\ C3:∥W_{\Omega }∥≽∥W_{E}∥,\\ C4:W_{\Omega }≼I_{N_{R}}+\frac{H_{1}Q^{\#}H_{1}^{H}}{\hat{\sigma_{i}^{2}}},\\ C5:W_{E}≼I_{N_{E}}+\frac{H_{E}^{1}Q^{\#}{H_{E}^{1}}^{H}}{\hat{\sigma^{2}}},\\ C6:\mathrm{Tr}\left(\Omega_{p}H_{1}Q^{\#}H_{1}^{H}\right)\geq E. \end{array}$$

After obtaining the value of $W_{\Omega }$, the value of $\Omega_{p}$ is calculated by rearranging the terms in (19). Thus, $\Omega_{p}$ is given by

(22) $$\Omega_{p}=diag\left(I_{N_{R}}-\left(W_{\Omega }-I_{N_{R}}\right)D_{i}^{-1}\right).$$

where $D_{i}=\frac{H_{1}Q^{\#}H_{1}^{H}}{\hat{\sigma_{i}^{2}}}$, and the term diag denotes the diagonal elements of the matrix. Here, the constraint *C*3 guarantees a positive value of the secrecy capacity, and the constraints *C*4 and *C*5 denote boundary conditions for the variables $W_{\Omega }$ and $W_{E}$ (since we are maximizing the objective, it is necessary to bound the values of the capacities, as they can become overbound, resulting in an unbounded problem). The values of the parameters *Q* and $\Omega_{p}$ are obtained by solving P2−A (17), P3−A (21) and (22).

### 3.2. Transmission Mode Pos-B

The problem P1−B (8) is a non-convex maximization problem; similar to the procedure shown in Section 3.1.1 and Section 3.1.2, we divide the problem into separate problems and solve for the transmit power matrix and the power-splitting ratio. The final reformulated problems for solving the maximization problem in (8) include the following procedure:

(23) $$\begin{array}{c} P2-B:\max_{r_{\Omega },r_{E}}\log_{2}∥r_{\Omega }∥\\ C1:\mathrm{Tr}({\left(H_{E}^{2}\right)}^{†}\left(\tilde{\sigma^{2}}\left(r_{E}-I_{N_{E}}\right)\right){\left({H_{E}^{2}}^{H}\right)}^{†})\leq P,\\ C2:r_{E}≽I_{N_{E}},\\ C3:\log_{2}∥r_{\Omega }^{*}∥+\mathrm{Tr}\left({\left(r_{\Omega }^{*}\right)}^{-1}\left(r_{\Omega }-r_{\Omega }^{*}\right)\right)\\ -\log_{2}∥r_{E}^{*}∥-\mathrm{Tr}\left({\left(r_{E}^{*}\right)}^{-1}\left(r_{E}-r_{E}^{*}\right)\right)\geq R,\\ C4:T_{EH}^{\prime }\geq E. \end{array}$$

Here, $r_{\Omega }$ is given by

(24) $$I_{N_{R}}+\frac{{\left(I_{N_{R}}-\Omega_{p}\right)}^{\frac{1}{2}}H_{2}QH_{2}^{H}{\left(I_{N_{R}}-\Omega_{p}\right)}^{\frac{1}{2}}}{\tilde{\sigma_{i}^{2}}}≜r_{\Omega }.$$

The variable $r_{E}$ is given by

(25) $$I_{N_{E}}+\frac{H_{E}^{2}Q{H_{E}^{2}}^{H}}{\tilde{\sigma^{2}}}≜r_{E}.$$

Similar to (13), we have a corresponding expression for denoting the harvested energy constraint:

(26) $$T_{EH}^{\prime }=\mathrm{Tr}\left(\Omega_{p}{\left({\left(I_{N_{R}}-\Omega_{p}\right)}^{\frac{1}{2}}\right)}^{-1}\left(\tilde{\sigma_{i}^{2}}\left(r_{\Omega }-I_{N_{R}}\right)\right){\left({\left(I_{N_{R}}-\Omega_{p}\right)}^{\frac{1}{2}}\right)}^{-1}\right).$$

By solving (23) and (25), we obtain the intermediate transmit power matrix $Q^{\#}$ from the solution variable $r_{E}$. Using the transmit power matrix $Q^{\#}$, we solve the upcoming problem P3−B to obtain the power-splitting ratio:

(27) $$\begin{array}{c} P3-B:\max_{w_{\Omega },w_{E},\Omega_{p}}\log_{2}∥w_{\Omega }∥\\ C1:w_{\Omega }≽0,\\ C2:w_{E}≽0,\\ C3:∥w_{\Omega }∥≽∥w_{E}∥,\\ C4:w_{\Omega }≼I_{N_{R}}+\frac{H_{2}Q^{\#}H_{2}^{H}}{\tilde{\sigma_{i}^{2}}},\\ C5:w_{E}≼I_{N_{E}}+\frac{H_{E}^{2}Q^{\#}{H_{E}^{2}}^{H}}{\tilde{\sigma^{2}}},\\ C6:\mathrm{Tr}\left(\Omega_{p}H_{2}Q^{\#}H_{2}^{H}\right)\geq E. \end{array}$$

Similarly, the expressions for $w_{\Omega }$ and $w_{E}$ are as follows:

(28) $$w_{\Omega }≜I_{N_{R}}+\frac{{\left(I_{N_{R}}-\Omega_{p}\right)}^{\frac{1}{2}}H_{2}Q^{\#}H_{2}^{H}{\left(I_{N_{R}}-\Omega_{p}\right)}^{\frac{1}{2}}}{\tilde{\sigma_{i}^{2}}},$$

And,

(29) $$w_{E}≜I_{N_{E}}+\frac{H_{E}^{2}Q^{\#}{H_{E}^{2}}^{H}}{\tilde{\sigma^{2}}}.$$

Thus, from (28), the value for $\Omega_{p}$ also changes, and it is given by

(30) $$\Omega_{p}=diag\left(I_{N_{R}}-\left(w_{\Omega }-I_{N_{R}}\right)d_{i}^{-1}\right).$$

Here, $d_{i}$ can be written as $d_{i}=\frac{H_{2}Q^{\#}H_{2}^{H}}{\tilde{\sigma_{i}^{2}}}$. After solving P2−B (23), P3−B (27) and (30), we obtain the values of the transmit power matrix *Q* and the power-splitting ratio. By solving the optimization problems of the two transmission modes, we can calculate the value of the harvested energy. The closed-form expression for the harvested energy at Bob for is calculated using

(31) $$\mathrm{Tr}(\Omega_{p}^{\#}HQ^{\#}H^{H}).$$

Using (31), the amount of harvested energy available is then calculated.

### 3.3. Algorithm Description

The secure resource allocation algorithm for transmission modes (when Bob is at Pos-A and Pos-B) based on the “divide-and-conquer” approach is summarized in Algorithm 1. The intermediate counter value $I_{c}\left(i\right)$ is defined as the collection of optimization variables $Q^{\#}$ and $\Omega_{p}^{\#}$ at iteration-*i*. At the beginning of the algorithm, we initialize the maximum number of iterations $L_{\max}$, the initial value of the intermediate counter $I_{c}\left(0\right)$, the transmit power budget *P*, and the desired secrecy capacity *R*. We also initialize a threshold ($T_{I_{c}}$) for the intermediate counter ($I_{c}$). The values of the optimization variables ($\Omega_{p}^{\#}$ and $Q^{\#}$) are computed based on the solutions of the problems P2−A and P3−A for Bob at Pos-A (the problems P2−B and P3−B are solved for Bob at Pos-B). The values of the optimization variables are stored in $I_{c}\left(i\right)$ (when the algorithm is in the *i*-th iteration). The difference between the current and previous values of $I_{c}$ is calculated to determine the threshold. Theoretically, we can conclude that the algorithm converges if the difference is less than or equal to the defined threshold $T_{I_{c}}$.

The expressions $Q^{\#}$ and $\Omega_{p}^{\#}$ are obtained by solving different problems [P2−A;P3−A] when Bob is at Pos-A and [P2−B;P3−B] when Bob is at Pos-B. Since we made the difference explicit in Algorithm 1, we excluded the use of multiple subscripts for the sake of conciseness and brevity.

**Algorithm 1** Secure resource allocation for the transmission modes Pos-A and Pos-B
1:**Initialization**: Choose $L_{max}$ along with *P*, *R* values and initialize the values of *i*, $I_{c}\left(0\right)$ and $T_{Ic}$;2:
**repeat**
Mode Pos-A: Calculate the value of *Q* using the Problem P2−A for a givenvalue of $\Omega_{p}$ and set *Q* as $Q^{\#}$;Mode Pos-B: Calculate the value of *Q* using the Problem P2−B for a givenvalue of $\Omega_{p}$ and set *Q* as $Q^{\#}$;Mode Pos-A: For the value of $Q^{\#}$, solve for the value of $\Omega_{p}$ using the Problem P3−A, and set $\Omega_{p}$ as $\Omega_{p}^{\#}$;Mode Pos-B: For the value of $Q^{\#}$, solve for the value of $\Omega_{p}$ using the Problem P3−B, and set $\Omega_{p}$ as $\Omega_{p}^{\#}$;
3:    Now calculate the value of harvested energyby (31) using the values of $Q^{\#}$ and $\Omega_{p}^{\#}$ for the corresponding modes and store in $I_{c}\left(i+1\right)$.;4:    Set i=i+1;5:**until** *i* value reaches $L_{max}$ or $I_{c}\left(i\right)-I_{c}\left(i-1\right)\leq T_{Ic}$6:Return $Q^{\#}$ and $\Omega_{p}^{\#}$ as the final optimal solution.


## 4. Results and Discussion

In this section, we present the simulation results to illustrate the trade-off between harvested energy and secrecy capacity for a single-user multi-antenna SWIPT system in the presence of a multi-antenna eavesdropper. The Matlab and CVX tools are used to obtain the following results. The simulation results are performed to evaluate the proposed algorithm when Bob is present at Pos-A and Pos-B. The channel fading states $H_{1}$ and $H_{2}$ are generated over 1000 channel realizations. The differences between $H_{1}$ and $H_{2}$ are based on the distance between Alice and Bob. We generated the channel state conditions by varying the distance of Bob as it moves away from Alice. For Pos-A, the distance between Alice and Bob is 30 m, while for Pos-B, the distance between Alice and Bob is 80 m. The eavesdropper is stationary, and its distance from Alice is assumed to be 20 m. Table 1 shows the essential sets of simulation parameters used to generate the results. The transmit power available at Alice is varied in the range of 5 to 40 dBm. The expressions for the harvested energy in the nonlinear EH models [3] are as follows:

(32) $$E_{nonlinear}=\frac{\frac{M}{1+\exp(-a\left(\mathrm{Tr}\left(\Omega_{p}^{\#}HQ^{\#}H^{H}\right)-b\right))}-\frac{M}{1+\exp(ab)}}{1-\frac{1}{1+\exp(ab)}}.$$

Here, *M* denotes the maximum energy harvested at the receiver when the EH circuit is saturated, and (a,b) denotes the circuit parameters for the nonlinear EH model considered. For the nonlinear EH model, the circuit parameters [a=6400,b=0.003] and M=0.024 are chosen as given in [3] and the harvested energy is then calculated, where the circuit parameters a,b and the maximum harvested energy *M* simulate the effects caused by the constraints such as current leakage and hardware sensitivity [3,20]. The noise at Bob is −30 dBm (when Bob is at Pos-A). A worst-case scenario is considered by assuming that the noise at Bob increases to 1 dBm (while at Pos-B). The noise at the eavesdropper is assumed to be −10 dBm.

### 4.1. Performance Analysis Based on Trade-Off between Harvested Energy and Secrecy Capacity

#### 4.1.1. Bob at Pos-A

**Figure 2:**
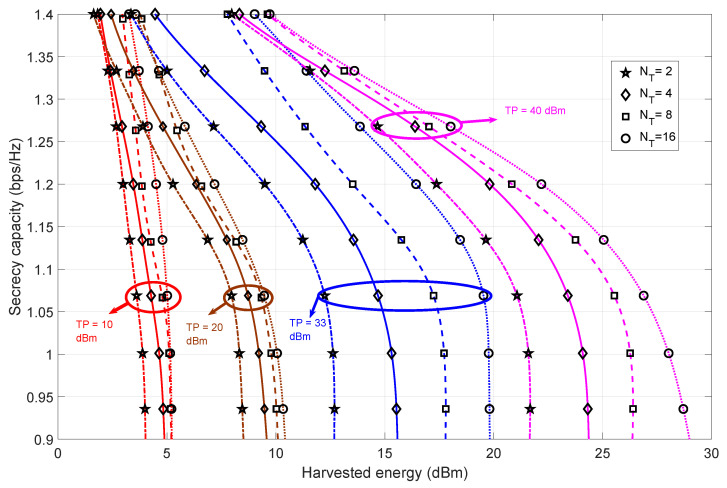
The trade-off between secrecy capacity and harvested energy for varying values of transmit antennas at Alice and transmit power when Bob is in Pos-A. The number of receiver antennas at Bob and eavesdropper are fixed at two.

In Figure 2, we present the trade-off between the secrecy capacity and harvested energy for varying transmit power and transmit antenna values at Alice. The simulations are performed when the channel fading state is $H_{1}$ and Bob is at Pos-A. The co-located SWIPT setting for Bob implies that the received signal is split between energy harvesting and information decoding. As the formulated problem is information capacity maximization, the solution to the variables $\Omega_{p}$ and *Q* are “fine tuned” so that the overall information capacity (thereby, the secrecy capacity) is maximized. This means that due to the maximization of the information capacity, there will be a decrease in the value of $\Omega_{p}$, which further decreases the value of the harvested energy (as $\Omega_{p}$ is directly proportional to harvested energy).

The achievable harvested energy values and corresponding range are small for the lower transmit power regimes due to the directly proportional relationship between the transmit power matrix, harvested energy and the transmit power constraint *C*1 in P1−A (7). However, the harvested energy range becomes wider as the transmit power value increases. This increase in range is due to the inherent properties of the transmit power matrix *Q*. Increasing the number of transmit antennas at Alice increases the matrix dimension of *Q*, which further amplifies the harvested energy (which can be verified from the harvested energy expression in (31)). Hence, we can infer that the increase in the number of transmit antennas is the least noticeable (due to the narrow range of harvested energy values) when the transmit power is low. Comparing within the higher transmit power regime, the increase in the harvested energy decreases the secrecy capacity (for all the combinations of transmit antennas at Alice). Overall, the trend of the secrecy capacity is steep. This is due to the more stringent constraint imposed by the formulated problem.

#### 4.1.2. Bob at Pos-B

**Figure 3:**
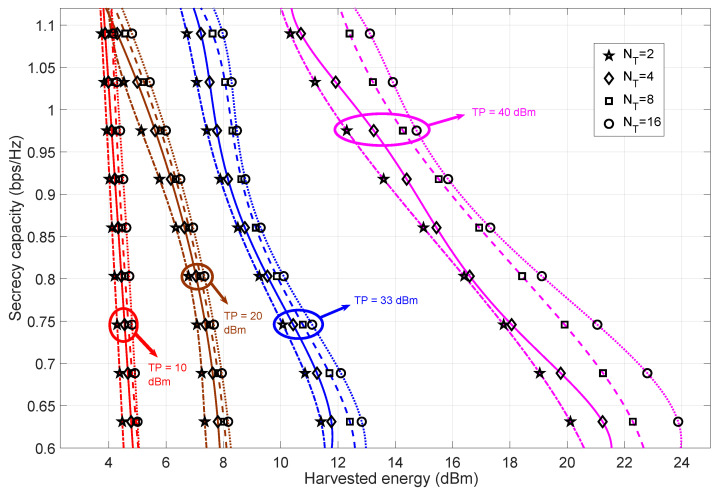
The trade-off between secrecy capacity and harvested energy for varying values of transmit antennas at Alice and transmit power when Bob is in Pos-B. The number of receiver antennas at Bob and eavesdropper are fixed at two.

Figure 3 illustrates the relationship between the secrecy capacity and harvested energy for different transmit power values and antennas. The analysis focuses on the channel fading state $H_{2}$, where Bob is moving away from Alice. Theoretically, this results in an overall decrease in the upper bound of both the secrecy capacity and the harvested energy. Also, increasing the harvested energy decreases the secrecy capacity values. We can conclude that the difference in harvested energy is significantly high for the high transmit power regime. In summary, increasing the transmit power increases the harvested energy values. We can also observe that increasing the number of transmit antennas at Alice has a smaller effect on the change in the harvested energy values. This difference in the consequences of varying the values of transmit antennas and transmit power can be attributed to the influence of the transmit power matrix on the proposed algorithm. The steepness of the trend of the trade-off is significantly high for the lower transmit power regime. From this observation, we can infer that the secrecy capacity decreases significantly as the harvested energy value increases. This is due to the lack of transmit power availability to maintain the required harvested energy due to the co-located nature of the SWIPT system.

#### 4.1.3. Comparison with Benchmarks

**Figure 4:**
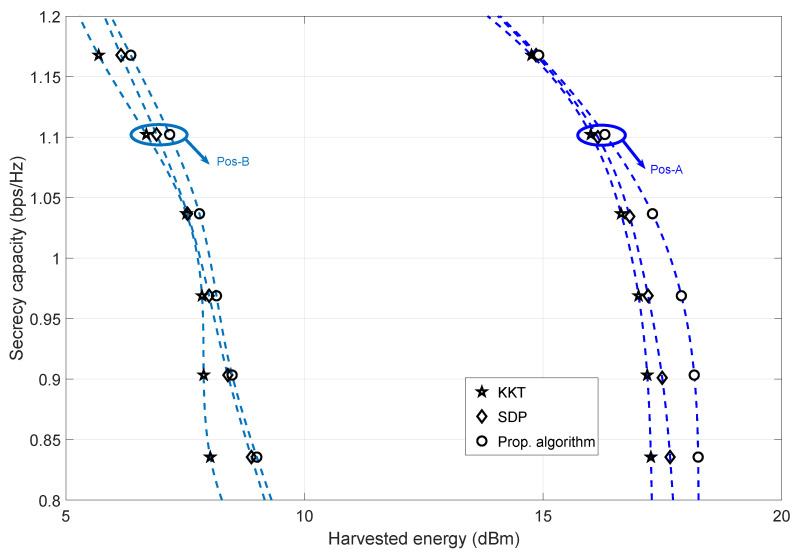
The trade-off between secrecy capacity and harvested energy for fixed value of transmit antennas (taken as 8) at Alice for Pos-A and Pos-B, analyzed for different algorithms—SDP [30] and KKT [36]. Here, transmit power is fixed at 33 dBm.

Figure 4 illustrates the secrecy capacity performance when the transmit power and transmit antennas (of Alice) are fixed for SDP, KKT, and the proposed algorithms. The harvested energy is computed by the solution variables *Q* and the power-splitting ratio $\Omega_{p}$ (which can be verified from (31)). We observe a steep decrease in the secrecy capacity values as the harvested energy increases. This is due to the co-located nature of Bob’s EH/ID. However, when comparing Pos-A and Pos-B, significant differences in the (achievable) harvested energy can be observed. This is due to the distance variation and channel fading state of Pos-A and Pos-B of Bob. We can also infer that to achieve positive secrecy capacity values between Pos-A and Pos-B, there is a significant drop in the harvested energy values between Pos-A and Pos-B. This phenomenon can be attributed to the information capacity maximization (of Bob), where our goal is to optimize the information capacity while maintaining the harvested energy and secrecy capacity requirements. Our algorithm successfully maintained the required secrecy capacity values by minimizing the harvested energy value (via the optimization of the $\Omega_{p}$ variable). That is, a decrease in $\Omega_{p}$ reduces the harvested energy, thereby maintaining the required secrecy capacity.

### 4.2. Analysis of Secrecy Capacity for Varying Number of Eavesdropper Antennas

#### 4.2.1. Bob at Pos-A

**Figure 5:**
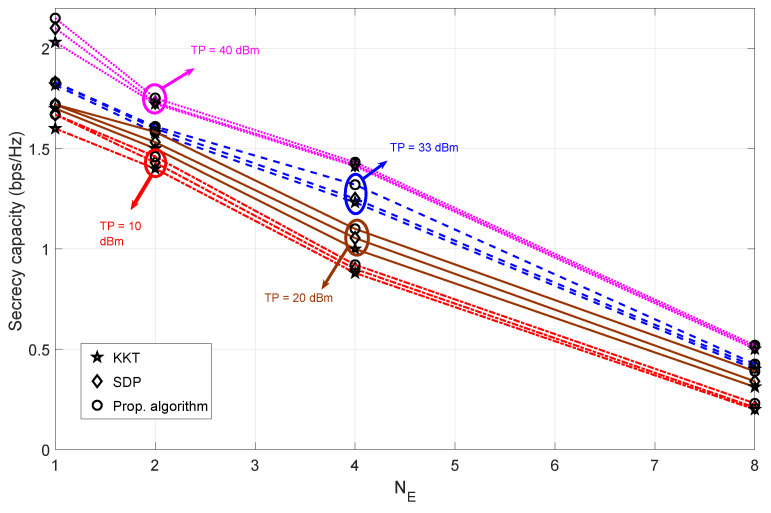
The trade-off between secrecy capacity for varying values of transmit power and eavesdropper antennas when Bob is in Pos-A. The number of antennas at the eavesdropper side is assumed to be varying $N_{E}$ = 1, 2, 4, 8. Algorithms compared are SDP [30] and KKT [36].

Figure 5 shows the secrecy capacity for different numbers of eavesdropper antennas. As the number of eavesdropper antennas increases, the secrecy capacity value decreases. This is because the information capacity value of the eavesdropper increases as the number of eavesdropper antennas increases (thus decreasing the secrecy capacity value). However, when the number of eavesdropper antennas is eight, the secrecy capacity barely exceeds the secrecy capacity of 0.5 bps/Hz. This means that simultaneously increasing the number of eavesdropper antennas and decreasing the transmit power has a negative effect on the secrecy capacity values. However, the increase in transmit power has a positive effect on the secrecy capacity values, resulting in an overall increase in the secrecy capacity (seen throughout the $N_{E}$ regime). The secrecy capacity difference for all the algorithms is almost the same for higher transmit power values. However, for lower values of transmit power, the differences in performance based on secrecy capacity can be observed. For all the simulated configurations of $N_{E}$, the proposed algorithm can achieve higher values of secrecy capacity, with a maximum difference in performance observed when $N_{E}$ = 4 and the transmit power is at 8 dBm.

#### 4.2.2. Bob at Pos-B

**Figure 6:**
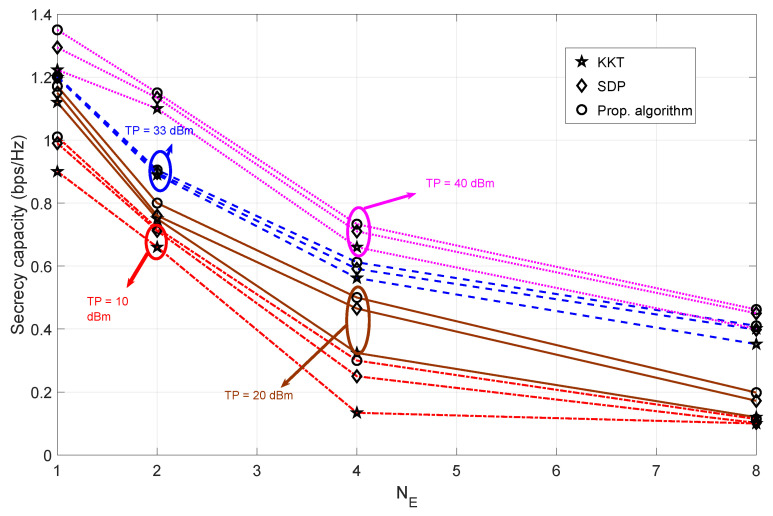
The trade-off between secrecy capacity for varying values of transmit power and eavesdropper antennas when Bob is at Pos-B. The number of antennas at eavesdropper side is assumed to be varying $N_{E}$ = 1, 2, 4, 8. Algorithms compared are SDP [30] and KKT [36].

Figure 6 shows the effect of the number of eavesdropper antennas on the secrecy capacity for varying transmit power values when Bob is at Pos-B. The cause of the overall decrease in secrecy can be attributed to the increase in the resulting distance between Alice and Bob. This increase in distance reduces the value of the secrecy capacity (compared to the value of the secrecy capacity in Figure 5). Because of the change in Bob’s trajectory (as he moves further away from Alice), the beamforming technique is ineffective. This is evident from the fact that the secrecy capacity value does not exceed 1.5 bps/Hz, even at a transmit power of 40 dBm. The decrease in secrecy capacity means that the overall information capacity of the eavesdropper increases as the number of antennas on the eavesdropper side increases. Because of the directly proportional relationship between the transmit power and the information capacity of Bob, increasing the transmit power increases the value of the secrecy capacity. This is true for all algorithms. The significant performance difference between different algorithms is observed when the transmit power is 8 dBm. This means that our proposed algorithm can maintain a relatively high secrecy capacity value, even when the transmit power is reduced.

### 4.3. Non-Zero Secrecy Capacity Probability Analysis

#### 4.3.1. Bob at Pos-A

**Figure 7:**
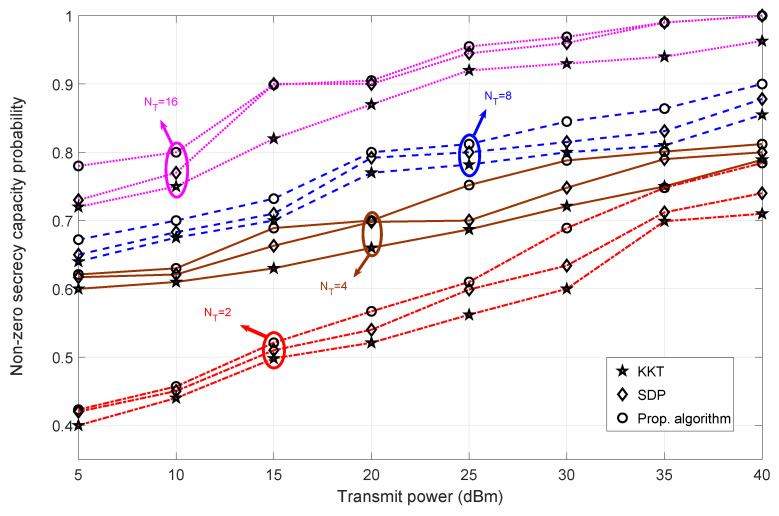
Analysis of non-zero secrecy capacity probability versus transmit power for varying transmit antennas at Alice when Bob is at Pos-A for different algorithms—SDP [30] and KKT [36].

Figure 7 shows the relationship between the non-zero secrecy capacity probability and the transmit power when Bob is at Pos-A. The non-zero probability of secrecy can be defined as the probability of achieving positive values of secrecy for the simulated set of iterations. The secrecy capacity requirement is relaxed [refer Appendix A], while the harvested energy requirement is increased to a limit (for a given transmit power). This analysis provides the probability of achieving positive secrecy capacity values for the proposed algorithm while maintaining a significant harvested energy requirement. The security aspect of the proposed algorithm is tested by maintaining a stricter harvested energy requirement, thereby accessing the number of iterations (we used 1000 iterations) where the secrecy capacity values fall below zero. We can conclude that the probability of obtaining a positive secrecy capacity increases as the transmit power increases. This is because the transmit power plays a significant role in achieving the more stringent harvested energy requirement and maintaining positive secrecy capacity values. The number of transmit antennas in Alice helps increase the probability of achieving positive secrecy capacity as shown in Figure 7. Therefore, to maintain non-zero secrecy capacity for a lower power regime, we must guarantee more transmit antennas for Alice. However, the difference in probability is not significant at a high transmit power.

Comparing different algorithms, our proposed algorithm maintains a higher probability value for the entire transmit power regime. However, for the higher transmit power regime, the probability of achieving positive secrecy capacity is almost the same for $N_{T}$ = 16. Overall, we can conclude that under a high transmit power regime, our proposed algorithm has a high probability of generating positive secrecy capacity values compared to other algorithms.

#### 4.3.2. Bob at Pos-B

**Figure 8:**
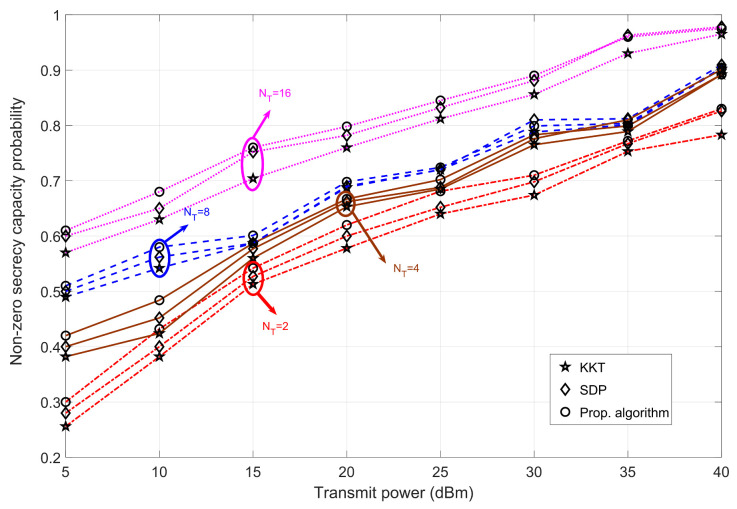
Analysis of non-zero secrecy capacity probability versus transmit power for varying transmit antennas at Alice when Bob is at Pos-B for different algorithms—SDP [30] and KKT [36].

Figure 8 illustrates the effect of transmit power on the achievable secrecy capacity for different values of transmit antennas. For a lower transmit power regime, the probability of achieving secrecy capacity is less than 0.7 for all combinations of transmit antennas. This is due to two reasons: one is the channel fading state $H_{2}$, which negatively affects Bob’s information capacity value, and the other is the inherent nature of low transmit power (which also reduces the value of secrecy capacity). As the transmit power increases, the probability of achieving a positive secrecy capacity increases for all transmit antenna configurations. We can observe that the difference between the probabilities of $N_{T}$ = 16 and other $N_{T}$ configurations is large. This suggests that to achieve positive secrecy capacity, it is only appropriate to deploy a large number of transmit antennas for Alice for a worst-case scenario (when the eavesdropper is in a better state than Bob). Unlike Figure 7 for Bob at Pos-A, the probabilistic analysis shows a clear difference in the simulated values for all the transmit power combinations considered when Bob is at Pos-B. This means that when Bob is farther away from Alice, our proposed algorithm can maintain a higher probability of achieving positive secrecy for the entire transmit power regime. For all $N_{T}$ combinations, the probability value differences between SDP and the proposed algorithm are small for higher transmit power values. However, for the lower transmit power regime, the difference in probability values between the different algorithms is relatively high. In summary, for higher values of $N_{T}$, the probability of achieving positive secrecy capacity increases.

### 4.4. Computational Complexity

The complexity analysis is performed for the proposed subproblems P2-A (17) and P3-A (21) (the computational complexity for Pos-A and Pos-B is identical; therefore, it is analyzed only for Pos-A). The increased computational complexity of the proposed algorithm is due to the presence of multiple matrices in the formulated problems. Table 2 shows the computational complexity of subproblems P2-A and P3-A, where $N_{T}$, $N_{E}$, and $N_{R}$ denote the number of antennas present at Alice, Eve, and Bob, respectively. The accuracy bounds for the proposed KKT and SDP algorithms are denoted as $\epsilon_{q}$, $\epsilon_{o}$ (for individual subproblems), $\epsilon_{q}\epsilon_{o}$, and ϵ, respectively. Table 2 gives an approximate upper bound on the complexity per iteration for the subproblems. Also, the computational complexity remains the same for both scenarios (when Bob is at Pos-A and when Bob is at Pos-B). There are special algorithms that can have lower complexity than the KKT algorithm. However, global optimality is not guaranteed when using such low-complexity algorithms. The interior-point approximation strategy can achieve low complexity, but this resource allocation strategy primarily leads to locally optimal solutions. Our proposed “divide-and-conquer approach” is a combination of sequential programming and a branch-bound strategy [38].

## 5. Conclusions

This paper investigates the trade-offs between secrecy capacity–harvested energy for the MIMO SWIPT system under two dynamic transmission modes in the presence of a multi-antenna eavesdropper. The system is modeled for co-located EH/ID receivers under the PS scenario. The optimization problems are formulated to compute the transmit power matrix and the power sharing ratio. The formulated problem has multiple variables in the product form; the problem is subdivided and solved individually to obtain respective solutions. Numerical results show the relationship between the secrecy capacity and the harvested energy. The performance of the proposed solution is studied for two transmission scenarios and compared with the algorithms available in the literature. The proposed solution shows a significant performance gain in terms of the secrecy capacity values for both transmission scenarios. Further research can be carried out with multiple multi-antenna legitimate receivers in the presence of eavesdropper(s). In addition, we can also explore resource allocation strategies to secure the SWIPT network in the presence of an active eavesdropper (thus extending the study beyond the passive eavesdropping scenario) for a more robust security framework for MIMO-SWIPT systems in vehicular networks.

## Figures and Tables

**Table 1:** Simulation parameters.

Parameters	Values
Number of transmit antennas at Alice	2, 4, 8, 16
Number of eavesdropper antennas	2, 4, 8
Noise Variance at Bob in Pos-A and Bob in Pos-B	−30 dBm and 1 dBm
Circuit Parameter value *a*	6400
Circuit Parameter value *b*	0.003
Transmit power values	5 to 40 dBm
Maximum harvested energy requirement	20 dBm
Path loss exponent	4
Rician factor	3 dB
Reference distance	10 m

**Table 2:** Computational complexities of the proposed, SDP and KKT-based algorithms.

Algorithms	Complexity Comparisons
SDP algorithm [30]	$O(N_{T}^{5}N_{R}^{3.5}N_{E}^{3}\log\left(\epsilon \right))$
KKT-based algorithm [36]	$O(N_{T}^{2}{\left(N_{R}N_{E}\right)}^{3}\log\left(\epsilon_{q}\epsilon_{o}\right))$
Proposed algorithm	P2-A: $O(N_{E}{\left(N_{T}N_{R}\right)}^{2}\log\left(\epsilon_{q}\right))$
	P3-A: $O(N_{T}N_{E}^{2}N_{R}^{3}\log\left(\epsilon_{o}\right))$

## Data Availability

Data sharing not applicable.

## Appendix A

The relation between the optimization variable *Q* and the channel fading state between Alice and Bob determines the secrecy capacity constraint and its variation over time. From constraint *C*4 in problems P1−A (7) and P1−B (8), we have the following secrecy constraint (for brevity, we discussed only P1−A):

(A1) $$\begin{array}{c} S_{C_{C}}=\log_{2}∥I_{N_{R}}+\frac{{\left(I_{N_{R}}-\Omega_{p}\right)}^{\frac{1}{2}}H_{1}QH_{1}^{H}{\left(I_{N_{R}}-\Omega_{p}\right)}^{\frac{1}{2}}}{\hat{\sigma_{i}^{2}}}∥\\ -\log_{2}∥I_{N_{E}}+\frac{H_{E}^{1}Q{H_{E}^{1}}^{H}}{\hat{\sigma^{2}}}∥ \end{array}$$

From (A1), we can observe that the $S_{C_{C}}$ depends on the variables *Q* and $\Omega_{p}$ as well as on the channel fading states $H_{1}$ and $H_{E}^{1}$. Thus, the positive values of the secrecy capacity are determined by the change between the states of *Q* and the channel states. Since Eve is relatively closer to Alice, we can assume that $\hat{\sigma_{i}^{2}}\geq \hat{\sigma^{2}}$. Let us assume

(A2) $$\alpha_{1}=\frac{∥H_{1}∥}{∥H_{E}^{1}∥},$$

and,

(A3) $$\alpha_{scc}=\frac{∥\hat{\sigma^{2}}{\left(I_{N_{R}}-\Omega_{p}\right)}^{\frac{1}{2}}H_{1}QH_{1}^{H}{\left(I_{N_{R}}-\Omega_{p}\right)}^{\frac{1}{2}}∥}{∥\hat{\sigma_{i}^{2}}H_{E}^{1}Q{H_{E}^{1}}^{H}∥}$$

The variation of the values of $\alpha_{1}$ and $\alpha_{scc}$ determines the “relaxation zone” of the secrecy capacity for the considered dynamic system. To achieve a positive secrecy capacity, it is always better to keep $\alpha_{scc}$ greater than one. However, to achieve high values of harvested energy for the co-located SWIPT setup, it is imperative to have high values of $\Omega_{p}$. This reduces Bob’s information capacity, resulting in a value between 0 and 1.
